# Programmable shunt valves with a “virtual off” for intrathecal chemotherapy delivery in children with high-grade CNS tumors and hydrocephalus

**DOI:** 10.1007/s00381-026-07311-y

**Published:** 2026-05-11

**Authors:** Rina Dvir, Yair Peled, Dror Levin, Ronit Elhasid, Shlomi Constantini, Manina Maja Etter, Jehuda Soleman, Valentina Pennacchietti, Ulrich-Wilhelm Thomale, Jonathan Roth

**Affiliations:** 1 Department of Pediatric Hemato-Oncology, Dana Children’s Hospital, Tel Aviv Medical Center, Tel Aviv University, Tel Aviv, Israel; 2 Department of Pediatric Neurosurgery, Pediatric Brain Center, Dana Children’s Hospital, Tel Aviv Medical Center, Tel Aviv University, 6 Weizman Street, 64239 Tel Aviv, Israel; 3 Department of Pediatric Neurosurgery, University Children’s Hospital of Basel, Basel, Switzerland; 4 Faculty of Medicine, University of Basel, Basel, Switzerland; 5 Pediatric Neurosurgery, Charité Universitaetsmedizin Berlin, Berlin, Germany

**Keywords:** Intrathecal, Chemotherapy, Programmable valve, Hydrocephalus, High-grade tumor, Pediatric brain tumor

## Abstract

**Purpose:**

Current chemotherapy protocols for treatment of embryonal brain tumors in children may recommend administration of intrathecal chemotherapy, either by a lumbar tap or via an Ommaya reservoir. Children with concurrent hydrocephalus and shunts may have subtherapeutic levels of chemotherapy in the CSF due to constant CSF drainage to extra-CNS compartments. We present our experience in delivery of chemotherapy to children via programmable valves.

**Methods:**

A retrospective analysis of children with CNS malignancies together with hydrocephalus treated with a shunt and a programmable valve (CERTAS™ Plus Programmable Valves—Integra Life Sciences, proGAV®—Miethke) was conducted.

**Results:**

Eighteen children up to 16 years of age (mean age 5 years) were included. Main pathologies included medulloblastomas (7) and atypical rhabdoid teratoid tumor (5). Each patient underwent 3–55 intrathecal injections (17 ± 14). One patient developed symptomatic hydrocephalus during the injection, which resolved with valve resetting. There were no infections, leaks, or major complications. One child required a wound revision due to exposure of the proximal catheter related to extremely thin skin. One patient experienced a “stuck” setting of the valve. Eight children are alive with no active disease, 25–80 months after shunt placement (52 ± 21). There were no late effects related to IT chemotherapy.

**Conclusions:**

Programmable ventriculoperitoneal valves are a safe method for delivery of intra-ventricular chemotherapy in children. This technique may potentially have an added value for children with concurrent shunts and may also obviate the need for an additional ventricular access device (such as an Ommaya reservoir).

## Introduction

Current protocols for high-risk embryonal and non-embryonal tumors (i.e., medulloblastoma and ATRT) advocate direct administration of chemotherapy to the ventricular system in addition to systemic chemotherapy. The conventional mode of administration is either by lumbar puncture or insertion of an Ommaya reservoir [1, 2]. IT (intrathecal) chemotherapy medications frequently include methotrexate, ARA-C, topotecan, and hydrocortisone. IT delivery may also be utilized for experimental therapies such as Car-T cell therapy, radio-immunotherapy, or antibiotics.

Typically, infants with high-grade tumors such as embryonal tumors have concurrent hydrocephalus and a ventriculoperitoneal shunt. When administering IT drugs with a shunt, some of the drugs will inevitably drift to the abdomen, thus lowering the CSF level of these drugs and reducing their efficacy [3]. Prior studies describing on-off or high-resistant valves used to enable IT injection while avoiding drug level reduction have been reported only rarely, and these valves were usually placed concurrently with an additional Ommaya reservoir for the IT injection [4–6]. Some valve systems were connected linearly with an Ommaya system [7]. Programmable magnetic ventriculo-peritoneal shunts were originally designed for better control of hydrocephalus by enabling valve pressure adjustments to optimize CSF drainage rate. Rarely have programmable valves been used for drug administration [3, 8].

In this multicenter international study, we report using programmable valves as a port for IT chemotherapy injection, as well as to temporarily decrease CSF drainage.

## Methods

This is an international multicenter retrospective study. Three centers were included: Dana Children’s Hospital, Tel Aviv Medical Center, Tel Aviv, Israel, Charité Universitaetsmedizin Berlin, Germany, and University Children’s Hospital of Basel, Basel, Switzerland. Following an IRB approval, we retrospectively collected data on children younger than 18 years of age with programmable valves that were used for IT treatment. Patient and caregiver approval were waived.

Two valves were used by these centers:

1. CODMAN® CERTAS® Plus Small Programmable Valve (Integra). This valve has 8 settings which may be set by an external magnetic device. Opening pressure settings range between 25 and 215 mmH_2_O, with an additional “virtual off” setting, set at an opening pressure of 400 mmH_2_O [9]. There are 2 subtypes—with and without a siphon-guard component.
2. proGAV® (Miethke). This valve has a programmable component (pressure settings of 0–20 cmH_2_O) and a gravitational unit (25 or 30 cmH_2_O). This valve does not have a “virtual off,” but at its highest setting, the resistance results in 200 mmH_2_O in the lying and 500 mH_2_O in the standing position. When using the proGAV® valve, a burr hole prechamber reservoir was added. Both valves are designed to withstand unintended pressure setting changes due to external magnetic influences, up to and including a 3-T MRI.

For both valves, the reservoir is placed proximal to the valve mechanism, and there is no unidirectional component between the reservoir and the ventricular catheter. Thus, there is minimal resistance between the reservoir and the ventricle when injecting media through it, and when the valve is set at maximal pressure settings (or to “virtual off”), the media is anticipated to solely reach the ventricle and not the distal component of the shunt.

### Intrathecal chemotherapy injection

Procedures were performed in an outpatient setting unless hospitalization was required for other reasons. Prior to administration of chemotherapy, the magnetic shunt is adjusted to the maximal setting (or “virtual off”) to minimize the CSF drainage, and remained closed for 1–4 h after injection. Maximal volume of injected fluid was 1–2 cc.

Using a sterile technique, the valve is tapped using a 23-G butterfly needle. A few cc of CSF are aspirated and sampled, followed by injection of the chemotherapeutic drug, then flushed by 1 cc of CSF. Children are discharged an hour after re-setting the shunt to the preprocedural baseline setting.

The goal of this study is to describe the safety of this technique, as well as patient outcomes.

### Statistical analysis

As this is a small series, the data is presented in a descriptive manner. Data is presented as means and standard deviation (SD). We conducted calculation of overall survival presented in form of a Kaplan-Meier curve. All calculations were done using Microsoft Excel. No statistical analyses are described.

## Results

Eighteen children were included in this study (10 females). The pathologies included medulloblastoma (*N* = 7), ATRT (*N* = 5), pineoblastoma (*N *= 2), metastatic Ewing sarcoma, metastatic rhabdomyosarcoma, infantile hemispheric glioma, and cerebral ALL (1 each).

Age range at tumor diagnosis was 1 week old–16 years old (5 ± 5.2 years). Seven were above the age of 3 years old (Table 1).

**Table 1:** Patient characteristics

*n*	18
female/male (*n*)	10/8
Age (mean±STD)	5±5.2 yrs
Infants (*n*)	11
Certas Plus (*n*)	7
proGAV (*n*)	11
Histology	Medulloblastoma: 7 ATRT: 5 Pineoblastoma: 2 Ewing: 1 Rhabdomyosarcoma: 1 Infantile hemispheric glioma: 1 CNS ALL: 1
Drugs administered (n)	Methotrexate (9) Topotecan (8) Etoposide (4) Cytarabine (4) Prednisone (5)
CNS metastasis (*n*)	13
IT injections (mean±STD)	3–55 (17±14)
Mortality (n/time)	8/7.8±5.8 months
Progressive disease (*n*/time)	2/10 and 100 months
Complete remission (*n*/time)	8/52±21 months

Each patient underwent 3–55 IT injections (17 ± 14). Injected drugs included methotrexate, topotecan, etoposide, cytarabine, and prednisone.

**Fig. 1:**
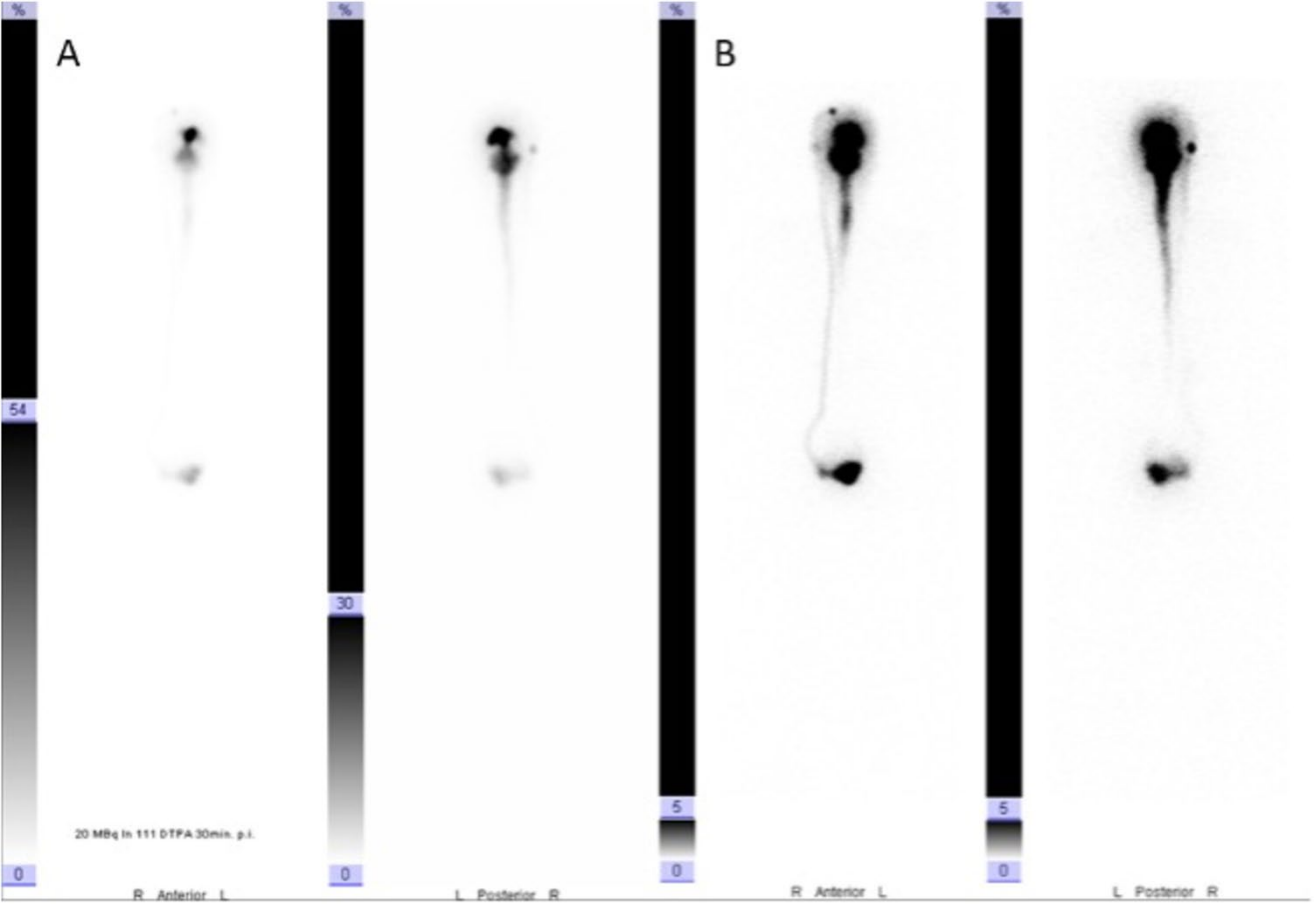
Seventeen-year-old girl with a recurrent medulloblastoma scheduled for IT Methotrexate administration (this case was not included in the study). Two milliliters of the tracer (22.6 MBq Indium-DTPA) were given in the right frontal reservoir of the VP shunt after the proGAV was adjusted to 20/50 cmH_2_O. The distribution of the tracer was investigated within the entire CSF spaces 30 min (**A**) and 1 h (**B**) following injection. A good distribution of the tracer was proven already at 30 min into the spinal canal. At 1 h, limited tracer activity was seen in the shunt system and the lower peritoneum compared to the CNS CSF spaces

In one patient, a CSF scintigraphy investigation was performed to prove feasibility of the concept and also to prove free CSF circulation in the individual case of a 17 y.o. girl with a recurrent medulloblastoma scheduled for IT Methotrexate administration. Therefore, 2 ml of the tracer (22.6 MBq Indium-DTPA) was given in the shunt reservoir after the proGAV was adjusted to 20/50 cmH_2_O following the same protocol as described above. The distribution of the tracer was investigated within the entire CSF spaces following injection. A good distribution of the tracer was proven at 30 min in the spinal canal after injection (Fig. 1).

There were no infections, leaks, or major complications. One child had somnolence and fever due to a viral disease. One child encountered valve-setting malfunction (CERTAS® valve was stuck at the setting of “6” with no clinical implications). He continued his IT injections despite this malfunction and is currently 4.5 years later with no active disease. One child required wound revision due to exposure of the proximal catheter, related to extremely thin skin. The same child at one point had clinical and radiological shunt over-drainage, solved by increasing the valve settings. In one case some of the intrathecal treatments had to be aborted since the child developed hydrocephalus symptoms; no change in regimen was done since this occurred rarely.

**Fig. 2:**
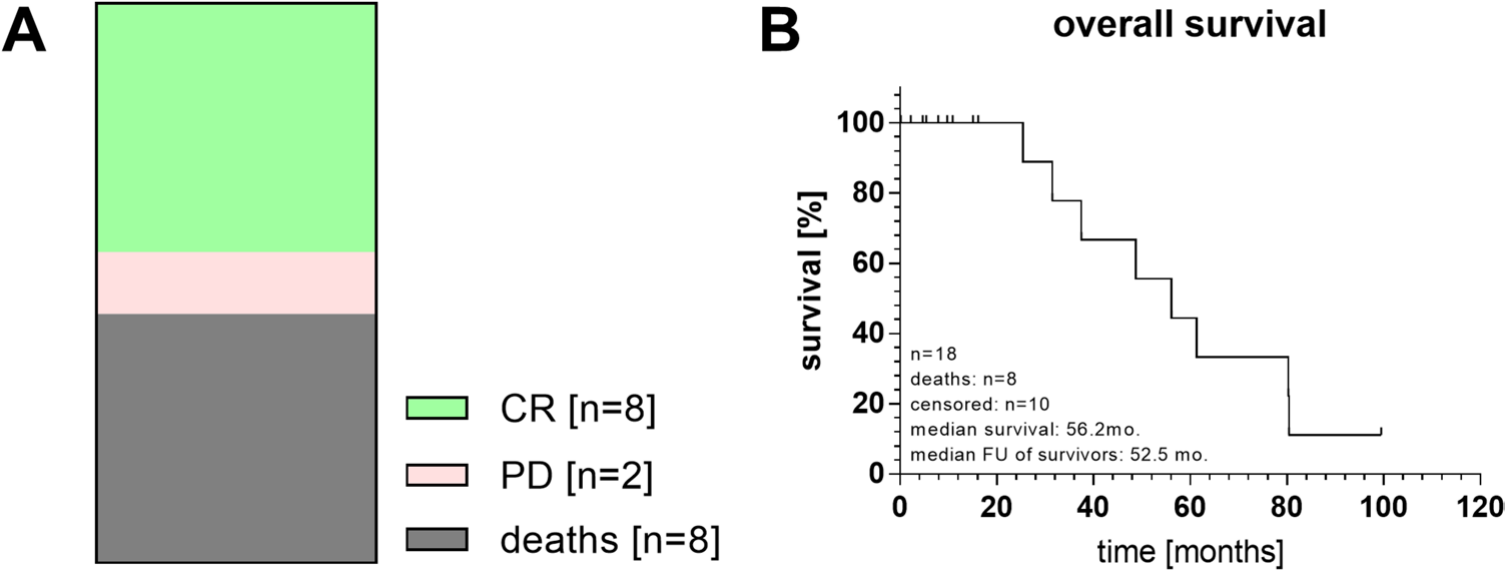
Patient outcomes (**A**) and Kaplan-Meier survival curve (**B**). CR complete recovery, PD progressive disease

Eight children died of their disease up to 16 months after the shunt placement (7.8 ± 5.8). Two children are alive with an active disease 10 and 100 months after shunt placement, and 8 children are alive with no active disease, 25–80 months after shunt placement (52 ± 21) (Fig. 2). There were no late effects related to IT chemotherapy.

## Discussion

We report on a small series of 18 children with concurrent high-grade CNS tumor and hydrocephalus, treated by IT CT injections to a programmable valve. Our results show that the technique is simple and safe. The most significant complication was 1 instance of the valve being “stuck” on one specific setting, with no ability to reprogram it. Despite the fact that the valve was stuck on a high-pressure setting, the child did not develop hydrocephalus. Although not of clinical significance in this case, and although we are unable to state the reason for this complication, it is important to note since it may pose significant risks. Possible causes of this malfunction may include being secondary to the external effect of an MRI system, or secondary to an internal effect, such as a small blood clot introduced during injection. Both the CERTAS® and proGAV® valves are intended to withstand external electromagnetic fields up to (and including) 3 Tesla. Nevertheless, these children often undergo repeated MRI scans, and thus, despite the safety profile of the valve, we cannot exclude this as a cause. Further, in one case, the child developed symptoms of hydrocephalus during a treatment session, which led to the premature termination of that session. As this occurred only rarely over the entire treatment course and the symptoms were mild, the overall treatment was not discontinued. Nevertheless, these treatments should preferably be conducted in an outpatient setting at a specialized center, with close monitoring—particularly during periods when the shunt is functionally closed, as this may precipitate acute hydrocephalus symptoms. Importantly, the occurrence of hydrocephalus symptoms during a single treatment session does not necessitate permanent discontinuation of therapy, as symptoms may not recur in subsequent sessions.

Burger et al. described 2 cases of children with an “on-off” valve who were undergoing injection of CT [5]. They closed the valve for 24 h following the injection to maximize CSF distribution of the drugs. Their system consisted of 1 intracranial catheter, with several parts, including a Rickham reservoir, in addition to the valve system. Czech et al. described 8 cases using an “on-off” system, which was closed for 2 h following the IT injection [10]. Kramer et al. increased a programmable valve setting for 6h following injections of radioimmunotherapy [8]. Zada et al. increased the valve pressure for 4h following injection and measured the distribution of a tracer injected concurrently with the chemotherapy [6]. Lin et al. have published a series on IT CT injection using an Ommaya connected to an “on-off” shunt system [7]. They closed the system for 2–6 h following the injection. However, one of the limitations of the “on-off” valves is the inadvertent closure of the valve, which may lead to life-threatening conditions [10, 11].

Over recent years, use of an “on–off” valve has decreased, replaced by magnetic programmable valves. McThenia et al. reported on 15 cases using 6 different magnetic programmable valve models, evaluating drug distribution to the peritoneum at 2–4 h and 24 h after injecting In-111-DTPA [3]. In their model, McThenia et al. increased the valve pressure to the maximal setting for about 4–5 h and compared the distribution of the marker at 2–4 h and 24 h after IT injection. Their findings suggest that there does exist some peritoneal spread, although the number of cases per valve model, as well as heterogeneity in pathology, valve settings, ages, and other variables, does not enable identification of any clear advantage of one model over another.

The current series describes using the valve as a port of injection, with no need for an additional Ommaya reservoir. As stated, most prior series have placed a separate injection port.

### Limitations

Our series is small and heterogeneous; therefore, we are unable to statistically prove the efficacy or safety of the proposed technique. The time period of valve closure was chosen arbitrarily (1–4 h); the pharmacokinetics of the drugs was not studied. It is therefore unknown how much of the drug level in the CSF was effective over time. In one case, however, we were able to show good distribution of the drug using a tracer. Despite increasing the valve setting to the maximal value (and “virtually” closing the valve for the CERTAS® model), drug distribution may not resemble that of IT drug distribution via a lumbar tap or an Ommaya injection, as these children have CSF pathway obstruction or malabsorption. Thus, the CSF bulk flow is not equal to that of a child with no hydrocephalus.

## Conclusions

Children with high-grade brain tumors and hydrocephalus with a VP shunt may benefit from the use of a programmable valve with a virtually off function, which may enable direct IT injection to the valve, sparing the need for an additional port, while temporarily reducing the valve function (or even “closing” it), reducing drug level reduction due to CSF shunting. This technique may be utilized even in small infants, with a high safety profile and minimal complications.

## Data Availability

No datasets were generated or analysed during the current study.

## References

[1] Peyrl A, Chocholous M, Azizi AA et al (2014) Safety of Ommaya reservoirs in children with brain tumors: a 20-year experience with 5472 intraventricular drug administrations in 98 patients. J Neurooncol 120:139–145. 10.1007/s11060-014-1531-1 25017328 10.1007/s11060-014-1531-1

[2] Kramer K, Pandit-Taskar N, Humm JL et al (2018) A phase II study of radioimmunotherapy with intraventricular 131I-3F8 for medulloblastoma. Pediatr Blood Cancer. 10.1002/pbc.26754 28940863 10.1002/pbc.26754 PMC6692907

[3] McThenia SS, Pandit-Taskar N, Grkovski M et al (2022) Quantifying intraventricular drug delivery utilizing programmable ventriculoperitoneal shunts as the intraventricular access device. J Neurooncol 157:457–463. 10.1007/s11060-022-03989-7 35403968 10.1007/s11060-022-03989-7 PMC11292655

[4] Palejwala SK, Stidd DA, Skoch JM et al (2014) Use of a stop-flow programmable shunt valve to maximize CNS chemotherapy delivery in a pediatric patient with acute lymphoblastic leukemia. Surg Neurol Int. 10.4103/2152-7806.139381 25225619 10.4103/2152-7806.139381 PMC4163905

[5] Burger MC, Wagner M, Franz K et al (2018) Ventriculoperitoneal shunts equipped with on-off valves for intraventricular therapies in patients with communicating hydrocephalus due to leptomeningeal metastases. J Clin Med. 10.3390/jcm7080216 30110924 10.3390/jcm7080216 PMC6111529

[6] Zada G, Chen TC (2010) A novel method for administering intrathecal chemotherapy in patients with leptomeningeal metastases and shunted hydrocephalus: case report. Neurosurgery. 10.1227/01.NEU.0000383138.78632.BA 20679917 10.1227/01.NEU.0000383138.78632.BA

[7] Lin N, Dunn IF, Glantz M et al (2011) Benefit of ventriculoperitoneal cerebrospinal fluid shunting and intrathecal chemotherapy in neoplastic meningitis: a retrospective, case-controlled study. Clinical article. J Neurosurg 115:730–736. 10.3171/2011.5.JNS101768 21721878 10.3171/2011.5.JNS101768

[8] Kramer K, Smith M, Souweidane MM (2014) Safety profile of long-term intraventricular access devices in pediatric patients receiving radioimmunotherapy for central nervous system malignancies. Pediatr Blood Cancer 61:1590–1592. 10.1002/pbc.25080 24777835 10.1002/pbc.25080

[9] Czosnyka Z, Pickard JD, Czosnyka M (2013) Hydrodynamic properties of the Certas hydrocephalus shunt: laboratory investigation. J Neurosurg Pediatr 11:198–204. 10.3171/2012.10.PEDS12239 23215818 10.3171/2012.10.PEDS12239

[10] Czech T, Reinprecht A, Dietrich W et al (1997) Reversible occlusion shunt for intraventricular chemotherapy in shunt-dependent brain tumor patients. Pediatr Hematol Oncol 14:375–380. 10.3109/08880019709041597 9211542 10.3109/08880019709041597

[11] Hertle DN, Tilgner J, Fruh K et al (2011) Reversible occlusion (on-off) valves in shunted tumor patients. Neurosurg Rev 34:235–242. 10.1007/S10143-010-0297-Y 10.1007/s10143-010-0297-y 21107629

